# The complete mitochondrial genome of the hybrid honey bee, *Apis mellifera capensis × Apis mellifera scutellata*, from South Africa

**DOI:** 10.1080/23802359.2016.1250132

**Published:** 2016-11-11

**Authors:** Amin Eimanifar, Rebecca T. Kimball, Edward L. Braun, James D Ellis

**Affiliations:** a Honey Bee Research and Extension Laboratory, Department of Entomology and Nematology, University of Florida, Gainesville, FL, USA;; b Department of Biology, University of Florida, Gainesville, FL, USA

**Keywords:** Hybrid honey bee, mitogenome, next-generation sequencing, *Apis mellifera capensis × Apis mellifera scutellata*, South Africa

## Abstract

We characterized the complete mitogenome sequence of the South African hybrid honey bee *Apis mellifera capensis* ×* Apis mellifera scutellata* using genome skimming. The mitochondrial genome was a circular molecule 16,340 bp in length with a gene organization identical to that of the other *A. mellifera* mitogenomes. The base composition is 43.2% A, 9.7% C, 5.6% G, and 41.5% T, with an A + T content of 84.7%. The mitogenome had 13 protein-coding genes (PCGs), 22 transfer RNAs, two ribosomal RNAs genes, and one control region. All PCGs were initiated by ATT, ATG, ATA, and ATC codons and were terminated by a TAA stop codon. The heavy strand encodes four PCGs, eight tRNAs, and two rRNAs. The light strand encodes nine PCGS and 14 tRNAs. A phylogenetic analysis of the PCGs reveals a close relationship between this hybrid honey bee and other *Apis* spp.

The modern diversity of *Apis* consists of nine species (Ruttner 1988), eight of the species being distributed naturally in Asia and, to a much lesser extent, the Middle East. The ninth *Apis* species, *A. mellifera* ? the western honey bee, is distributed in Europe. However, *A. mellifera* (western honey bee) includes 26 subspecies distributed worldwide (Ruttner 1988). These subspecies are adapted to many different environmental conditions (Danforth 2007). South Africa subspecies include *A.m. scutellata* (‘African’ bee) and *A.m. capensis* (cape bee) (Hepburn & Radloff 1998). Morphometric and molecular studies confirm a hybrid zone between the two subspecies (Hepburn & Radloff 1998).

We report the complete mitogenomic sequence of an *A.m. capensis* ×* A.m. scutellata* hybrid honey bee (accession number KX943034) collected from an apiary close to Beaufort West in South Africa (32°34′S – 22°64′E). Morphometric analysis confirmed the bee was a hybrid (unpublished data) and its colony falls within the hybrid zone (Hepburn & Radloff 1998).

Total genomic DNA was isolated using cetyltrimethylammonium bromide (CTAB) followed by phenol-chloroform-isoamyl alcohol (25:24:1) (see Hunt & Page 1995). Genomic DNA was quantified using a Qubit^®^ 3.0 Fluorometer (Thermo Scientific Inc., Waltham, MA). Genome skimming (Straub et al. 2012) was performed using pair-end sequencing (2 × 100 bp) on the Illumina HiSeq 2000 (San Diego, CA) sequencing platform.

FASTQ reads were mapped to *A.m. ligustica* (L06178.1) using Geneious R9.1.5 (Kearse et al. 2012). We followed the methods outlined by Eimanifar et al. (2016), which involve several iterations and sub-assemblies that are combined together. A final mapping of the reads was done to ensure that reads mapped without error.

The circular mitochondrial genome of the hybrid bee was 16,340 bp. It contained 13 protein-coding genes, 22 tRNAs, two rRNAs, and an AT-rich control region, matching the organization of *A.m. ligustica*. There was a strong A + T bias (84.7%). The heavy strand encoded four PCGs, eight tRNAs, and two rRNAs, while the light strand encoded nine PCGs and 14 tRNAs. Start codons included ATT, ATG, ATA, and ATC, while TAA (or a T that could be polyadenylated) was the only stop codon.

Since hymenoptera mitochondria may recombine (Gotzek et al. 2010; Mao et al. 2014), we compared this mitochondrion with *A.m. scutellata* (KJ601784) and *A.m. capensis* (KX870183). The genetic distance between the hybrid and *A.m. capensis* was 0.0015 while between the hybrid and *A.m. scutellata* was 0.0033. The three mitogenomes exhibited six codon differences: two united the parental subspecies, one united the hybrid with *A.m. scutellata*, and three united the hybrid with *A.m. capensis*. There were more substitutions at synonymous sites in PCGs or in RNAs that united the hybrid with *A.m. capensis* (19 and 7, respectively) than with *A.m. scutellata* (3 and 3, respectively). Additional differences in non-coding regions showed no consistent pattern. Overall, the hybrid bee exhibited greater similarity to *A.m. capensis* than to *A.m. scutellata* and there was no clear evidence for recombination (though our data could not exclude that possibility).

Using 13 mitochondrial protein-coding genes and two rRNAs, we estimated the ML tree with RAxML 8.0.20 (Stamatakis 2006) and the GTRGAMMA model using 10 random additions and 1000 bootstrap replicates (Figure 1). This revealed a close relationship between the hybrid bee and the *A.m. capensis*, and these taxa were sister to *A.m. scutellata*. The complete mitogenome of this hybrid bee is a first step towards understanding hybridization among *A. mellifera* subspecies.

**Figure 1.**
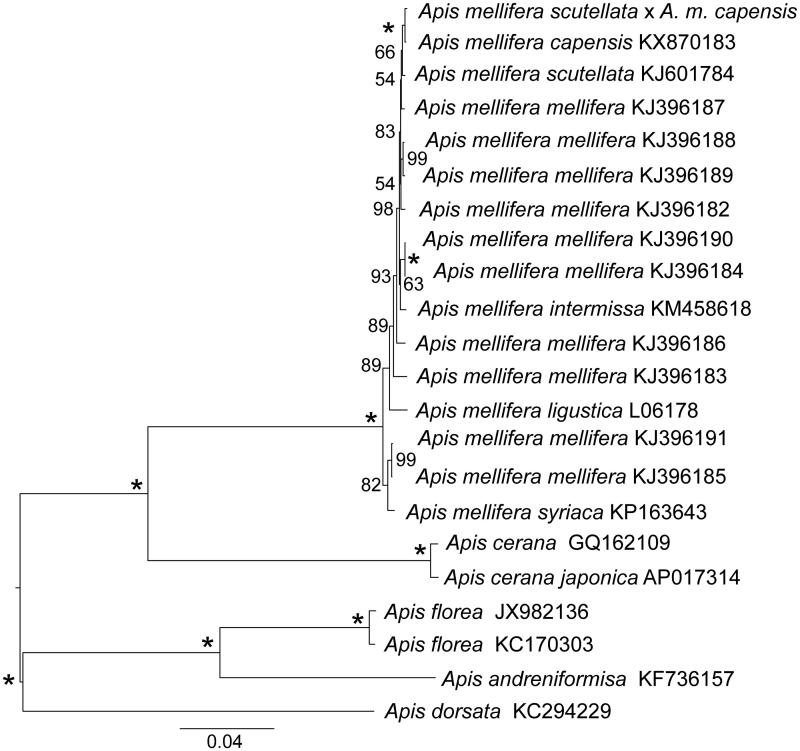
Phylogenetic tree analysis (ML topology) within the genus *Apis* based on the mitochondrial genome nucleotide sequence of the 13 protein-coding genes and two rRNAs. Midpoint rooting was used. The numbers next to each node (left side) represent the bootstrap values. The letter/number combination on the right side of each name indicates the GenBank accession numbers.
